# Methodological redundancy vs. clinical rigor: deconstructing causality claims in pediatric circadian and sedentary health trends

**DOI:** 10.1097/MS9.0000000000005403

**Published:** 2026-08-26

**Authors:** Sohail Memon

**Affiliations:** Liaquat University of Medical & Health Sciences (LUMHS), Jamshoro, Sindh, Pakistan

*Dear Editor,*

We read with great interest the recent editorial by Ahmed *et al* evaluating the dual threats of circadian rhythm disruption and screen-based sedentary behavior on pediatric growth and long-term metabolic health outcomes^[^1^]^. While the authors successfully showcase the clinical urgency of these rising technological lifestyle patterns, their diagnostic and policy conclusions demand a more rigorous physiological and methodological critique^[^1^]^.

Differentiating primary screen-induced circadian phase delays from underlying neurodevelopmental variations or socio-environmental confounding factors presents a uniquely challenging paradigm for modern pediatric health^[^1^]^. As demonstrated in the editorial’s discussion on sleep dysregulation, the downstream biochemical shifts, such as blunted nocturnal melatonin synthesis and altered growth hormone secretion profiles, exhibit an absolute structural overlap with conditions triggered by chronic academic stress or modern shift-work family structures^[^1,2^]^. Consequently, concluding a definitive causal linkage based purely on temporal digital media exposure is highly problematic^[^1^]^. The absence of an objective, validated, multi-system tracking framework makes it impossible to systematically isolate true device-driven blue light toxicity from broader socioeconomic dependencies, irregular nutritional timing, or metabolic changes linked to early-morning school schedules^[^1,3^]^.

Furthermore, while descriptive tracking confirms that technology acts as a primary driver of inactivity, relying on standalone smartphone applications or gamified augmented reality tools like Pokémon Go to fix the issue creates a major operational contradiction^[^1^]^. As the authors rightly acknowledge, initial behavioral gains in daily step counts face high attrition rates, rendering their long-term health sustainability highly uncertain^[^1,4^]^. Without a standardized clinical matrix, cumulative behavioral and metabolic organ stress can easily be misattributed to simple screen time rather than looking at total daily physical education infrastructure and sleep hygiene failures^[^1,3^]^. To realistically bridge this gap in routine, fast-paced pediatric practice, we propose a device-level, system-integrated workflow^[^1^]^. Instead of relying on manual parental counseling or unsustainable apps, pediatric screening should integrate automated operating system constraints that shift display light spectra at sunset and hard-lock entertainment interfaces at designated bedtimes^[^1,4^]^. Implementing this structured, low-burden lifestyle protocol ensures that clinical recommendations maintain the structural safety rigor required for evidence-based medicine.

Sincerely,

Sohail Memon

## Ethical approval

Not applicable.

## Consent

Not applicable.

## Data Availability

Not applicable.
